# Development and validation of the adolescent behavioural change Counselling Assessment Tool in Indonesia

**DOI:** 10.1186/s12913-024-10582-3

**Published:** 2024-02-28

**Authors:** Fransisca Handy Agung, Rini Sekartini, Nani Cahyani Sudarsono, Aryono Hendarto, Retno Asti Werdhani, Sri Retno Pudjiati, Lathifah Hanum, Affan Naufal, Susan M Sawyer

**Affiliations:** 1 Faculty of Medicine, Universitas Pelita Harapan, Jl. Jend. Sudirman No.20, Bencongan, Kelapa Dua, 15810 Tangerang, Banten, Indonesia; 2 Department of Child Health, Faculty of Medicine Universitas Indonesia - Cipto Mangunkusumo Hospital, Jl. Salemba Raya No.6 Jakarta Pusat, 10430 Jakarta, Indonesia; 3 Department of Community Medicine, Faculty of Medicine, Universitas Indonesia, Jl. Salemba Raya No.6 Jakarta Pusat, 10430 Jakarta, Indonesia; 4 Faculty of Psychology, Universitas Indonesia, Kampus UI, Depok, West Java Indonesia; 5 Balaraja Distric Hospital, Jl. Rumah Sakit No 88, Balaraja, Tangerang, Banten, Indonesia; 6 Centre for Adolescent Health, Royal Children’s Hospital and Murdoch Children’s Research Institute, Department of Paediatrics, The University of Melbourne, 50 Flemington Rd, 3052 Parkville, VIC Australia

**Keywords:** Adolescent, Behaviour-change, Counselling skill, Health professionals, Non-communicable diseases, Quality

## Abstract

**Background:**

Primary care provides an important context to engage adolescents and their families in healthy lifestyles with the goal of reducing future behaviour-related health problems. Developing a valid tool to assess health professionals’ skills in behavioural change counselling is integral to improving the quality of clinical care provided to adolescents in Indonesia.

**Methods:**

This work was nested within a project to develop a training program to enhance the behaviour-change counselling of adolescents and their parents by Indonesian primary care professionals. Initial item development was based on the content of the training module and the domain structure of the Behavioral Change Counselling Index (BECCI), a commonly used tool to assess counselling quality in healthcare settings. Expert panels were used to test content validity, while face validity was assessed by a group of trained psychologists. Inter-rater agreement was calculated prior to tests of construct validity and reliability, which involved psychologists rating 125 audio-taped counselling sessions between the health professional and adolescent patients, together with a parent.

**Results:**

An initial 13-item tool was developed using a 1–5 Likert scale. Validity and reliability testing resulted in the decision to use a 14-item tool with a 0–3 Likert scale. The scale was found to have a Cronbach’s α coefficient of 0.839 (internal consistency), and there was strong inter-rater agreement (0.931).

**Conclusion:**

The assessment tool known as the Adolescent Behavioural Change Counselling Assessment Tool, is a valid and reliable instrument to measure Indonesian health professionals’ behavioural-change counselling skills with adolescent patients. The tool provides an evaluation framework for future interventions that aim to improve health professionals’ skills in addressing adolescent behaviour-related health problems.

## Introduction

Adolescence is a time of great developmental opportunity, shaped by socioeconomic resources, education, cultural norms, family values, and peer contexts. These same factors influence behavioural patterns which contribute to much adolescent morbidity and mortality. This includes behaviours that contribute to unintentional injury, exposure to violence, sexual health outcomes including sexually transmitted infections and unplanned pregnancy, smoking and substance use, unhealthy eating habits and sedentary lifestyles, each of which carry risks to adolescents’ health and well-being [1, 2]. These behaviours also contribute to adult health. For example, each year 15 million people die prematurely between the ages of 30-69-years-old from non-communicable diseases (NCDs), with over 85% occurring in low- and middle-income countries (LMICs) [3]. In Indonesia, NCDs contribute a major proportion of the country’s adult health burden [4]. 

Risky behaviours can be identified, managed and monitored [1]. Behaviour counselling interventions have been found to be associated with reduction in risky behaviours without evidence of unintended harmful effects [5, 6]. Despite recognition of the significant short- and long-term impacts of behaviour-related health problems, and the availability of effective interventions, only about a third of adolescents with a diagnosable behaviour-related health disorder receive appropriate care [7], consistent with evidence that counselling interventions to address behaviour-related health problems are underutilized in healthcare settings [7, 8]. 

Many health professionals are poorly prepared to provide counselling to address behaviour-related health problems, especially in LMICs [8, 9]. In 2018, a national review in Indonesia revealed that poor counselling quality was a major weakness within health services for adolescents [10]. One approach to improve the identification and treatment of behaviour-related health problems within the health care system is to integrate behavioural health services into medical settings [7]. In Indonesia behaviour-change counselling techniques have not yet been included in the majority of health professional training curricula [11, 12] and there are no available tools to assess counselling quality. We recently developed a training program to promote the capacity of primary care providers to provide behavioural-change counselling to adolescent patients. In this paper, we describe the development and validation of an assessment tool, the Adolescent Behavioural Change Counselling Assessment Tool (ABC-CAT) to evaluate the effectiveness of the clinical training.

## Materials and methods

### Setting and context

An internet-based training to improve health professionals’ counselling skills was developed as an initiative to provide more accessible training on adolescent health within primary health services in Indonesia. The clinical training module was designed in three stages. Firstly, we undertook a literature review using a series of keywords such as weight management counselling, obesity prevention, behaviour change counselling, motivational interviewing (MI) and parenting in adolescents. Secondly, we undertook a qualitative study of adolescents and parents from different socio-demographic backgrounds in three provinces of Indonesia to explore barriers to healthy eating in the home [13]. These two steps informed the development of the initial training material. In the third step, we engaged with relevant professional organisations in Indonesia, the Ministry of Health (Republic of Indonesia) and individual adolescent health practitioners to review and refine the training material.

The final training module comprised sections on adolescent growth and development, healthy lifestyles, parenting, how to start a counselling session with adolescent patients, psychosocial screening, behaviour change principles and stages, motivational interviewing, and how to work with parents to promote their ability to foster their children make healthy choices. The development of the training material used constructive alignment theory, an integrative design for teaching in which the alignment between intended learning outcomes, teaching and learning activities, and assessment tasks is emphasized [14]. 

The assessment tool was developed to evaluate the counselling skills that were taught as part of this training program. Although the training was explicitly aimed at obesity prevention, the principles of the behaviour-change counselling skills were intended to be applicable to any behaviour-related health problem. The training and the assessment tool were developed in the Indonesian language (*Bahasa Indonesia*).

### Item development

**Fig. 1:**
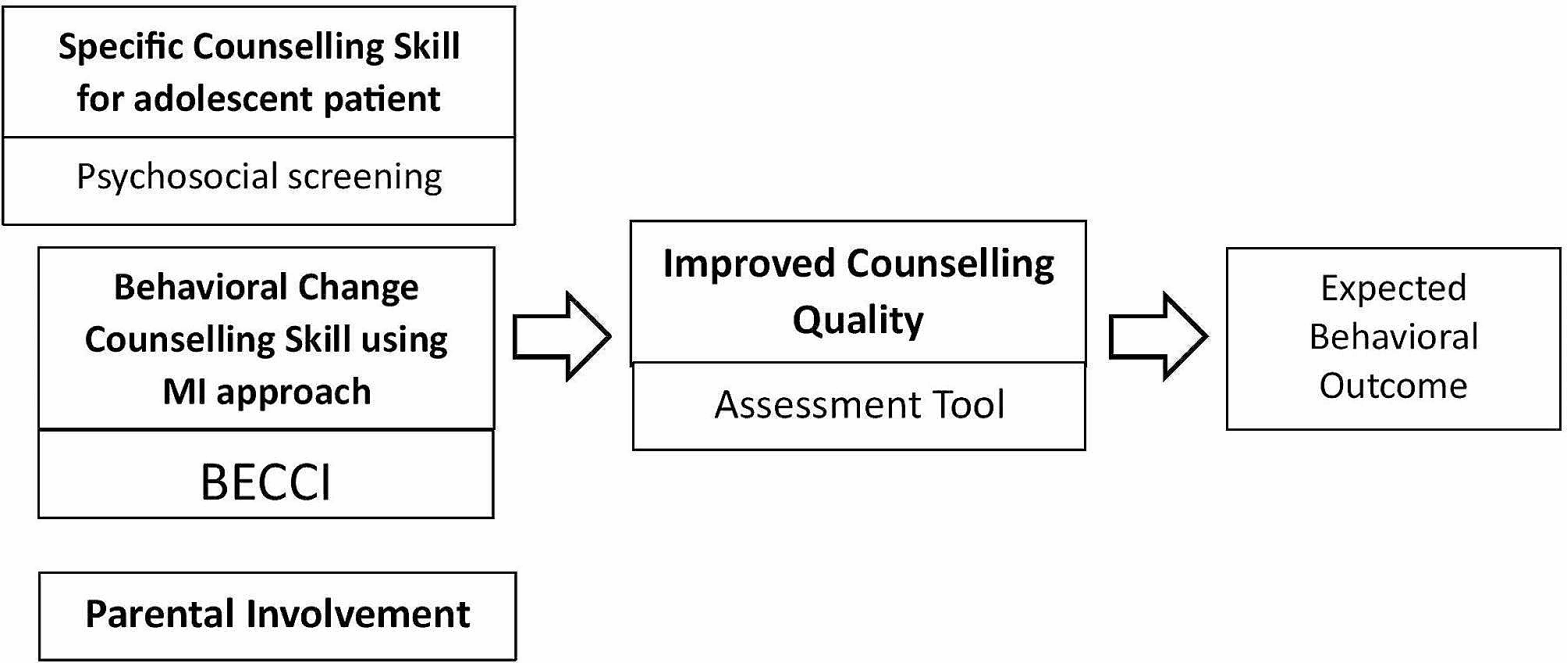
Conceptual framework for construct development of the assessment tool

The first step in the development of the assessment tool was to identify the key aspects of behaviour-change counselling skills that were taught within the training material [14]. The training was based on motivational interviewing (MI), a specific behaviour-change counselling approach that is widely used within health services, including for adolescent patients in primary care [15–17]. A number of assessment tools have been developed to assess the quality of counselling following MI training. Among the most widely used is the Behavioral Change Counselling Index (BECCI) which has been shown to have a Cronbach α-coefficient of 0.71 [18–20].. We initially created a 13-item measure that used the 11-item BECCI for items related to MI counselling techniques, to which we added two items that corresponded to the more adolescent-specific aspects of the training (psychosocial screening and parental involvement). All questions used a Likert scale response rating of 1–5. The conceptual framework for the development of the assessment tool and its constructs is shown in Fig. 1.

### Validity

A questionnaire requires tests of validity and reliablity to evaluate its accuracy and consistency. Validity expresses the degree to which the measurement of something (in this case, a survey or questionnaire) measures what it purports to. We assessed two major types of validity: content validity and construct validity. Content validity is the extent to which a questionnaire includes the most relevant and important aspects of a concept in the context of a given measurement application [21]. Face validity, an aspect of content validity, is the ability of an instrument to be understandable and relevant to the targeted user [21, 22]. Construct validity is the degree to which an instrument measures the theoretical construct that it is intended to measure. Reliability concerns the extent to which the measurement of a phenomenon provides stable or consistent results [21]. 

#### Content validity

Content validity was undertaken by an expert panel of 16 people that consisted of: representatives of the Indonesian Pediatric Society, the Indonesian Family Doctors Association, and the Indonesian Clinical Psychologist Association; six trained adolescent health care providers (physicians and nurses); two clinical psychologists (one child and adolescent psychologist and one MI trained psychologist); two paediatricians (one with particular expertise in child growth and development and one with specific expertise in nutrition and metabolic diseases); and one sports medicine specialist. Each member of the expert panel scored (0–4) the appropriateness of each item in the context of the training module, which provided the content validity index (CVI). Focus group discussions were then conducted with the expert panel, in which each item was discussed with the goal of improving the overall assessment tool.

#### Face validity

Face validity was assessed by the ability of the ABC-CAT to be understandable and relevant for clinical psychologists, who we used to evaluate the tool. In Indonesia, clinical psychologists are the only health professionals trained on MI. Using the Indonesian young clinical psychologist network, we recruited eight clinical psychologists who had been trained in MI. The clinical psychologists used the assessment tool to rate audiotaped consultations of counselling sessions of primary care professionals and adolescent patients and their parents. The intention of the validation was to confirm that there was clear understanding of each item and each assessment description by this group of professionals. Previously, the psychologist team had been given access to the online training modules and all training materials on the training website. After discussing the module content and the pedagogical intention of the training, the psychologist team conducted a reading test of the ABC-CAT. Following this, they individually used the assessment tool to evaluate the same two audio-taped counselling sessions. These then formed the basis of focus group discussions to refine the assessment tool [22]. 

#### Construct validity and internal reliability

We completed an inter-rater reliability test to ensure that there was acceptable agreement between all raters (the eight trained clinical psychologists) for each of the assessment items. For this purpose, an inter-class correlation coefficient (ICC) test was conducted using a sample size of 15 audio-taped counselling sessions, based on the simplified Winer and Walter formula which is sufficient to detect a strong level of agreement (above 0.7) [23]. Construct validity was established by corrected item-total correlation analysis of a sample size of 125, which was obtained from audio-taped counselling sessions of adolescent health practitioners from 17 provinces across Indonesia [24]. Items with a correlation coefficient less than 0.3 were omitted [25]. The internal consistency (reliability) was then examined using the Cronbach α coefficient, for which a coefficient of 0.7 or higher is considered reliable [25]. 

### Ethics

The Health Research Ethics Committee - Faculty of Medicine Universitas Indonesia and Cipto Mangunkusumo Hospital approved this study (approval number 829a/UNZ.F1/ETIK/PPM.00.02/2021). All participants gave written informed consent prior to their participation.

## Results

### Content validation

All 16 experts gave a score of 3 or 4 (appropriate or very appropriate) for the 13 assessment items which resulted in a total CVI score of 1. Discussion of each item within the subsequent focus groups resulted in two major changes. Firstly, a new item was added, namely “provided adolescents with the opportunity to be seen alone and made a confidentiality statement”. Secondly, the original Likert 1–5 scale was replaced with a simpler 0–3 scale. Finally, minor changes to the explanation for each score on each assessment item were made to more clearly differentiate a continuum of performance levels.

This process resulted in the assessment tool (ABC-CAT version 2.0) having 14 items across four sub-themes. The four sub-themes are the opening of a session, psychosocial screening, the specific behaviour-change counselling approach (MI) and parental involvement (see Table 1).

**Table 1:** The domain structure of the BECCI^*^ and ABC-CAT**

No	BECCI Assessment Item	BECCI Assessment Domain	ABC-CAT Assessment Domain
1	Practitioner invites the patient to talk about behaviour change	Opening session and permission seeking	Asks (1) how is she/he doing and (2) a brief history of any concern (1–3 questions) AND (3) explains how the session will be conducted
2	Practitioner demonstrates sensitivity to talking about other issues*		
3	Practitioner encourages patient to talk about current behaviour or status quo	Why and how of behaviour change	Communication skills: Open-ended questions, affirmation, reflective listening, and use of summaries MI counselling stages: engaging, focusing, evoking Provides Information: elicit - provide– elicit
4	Practitioner encourages patient to talk about behaviour change		
5	Practitioner asks questions to elicit how patient thinks and feels about the topic		
6	Practitioner uses empathic listening statements when patient talks about the topic		
7	Practitioner uses summaries to bring together what the patient says about the topic		
8	Practitioner acknowledges challenges about behaviour change that the patient faces	The whole counselling	
9	When practitioner provides information, it is sensitive to patient concerns and understanding**		
10	Practitioner actively conveys respect for patient choice about behaviour change		
11	Practitioner and patient exchange ideas about how the patient could change current behaviour	Discussion about target	Planning
12			New domain: Adolescent and Parents Introduction: greets the adolescent and the parents, then introduces self
13			Provides private space for the adolescent and a confidentiality statement
14			Involves parents in counselling

*Behavioral Change Counselling Index (BECCI)

** Adolescent Behaviour Change Counselling Counselling Assesment Tool (ABC-CAT)

Following the face validation process with the group of eight clinical psychologists, revisions were made to four items. Further revisions were also made to each of the assessment descriptions for each score for all 14 items. The reading level assessment also led to some simplification of language. This final assessment tool (ABC-CAT version 3.0) (see Table 2) then underwent tests of construct validity and internal reliability.

**Table 2:** Final scoring rubric for the Adolescent Behaviour Change– Counselling Assessment Tool (ABC– CAT) and inter-item correlation score for each item

	Rating Items	0	1	2	3	Inter-item correlation
Opening						
1	Greeting: greets the adolescent and parents (AND introduces themselves)	No greeting	Only greets the adolescent OR the parents	Greets the parents THEN greets the adolescent (AND introduces themselves)	Greets the adolescent first and THEN the parent (AND introduces themselves)	0.192
2	Introduction: (1) asks for news or the chief complaint OR (2) takes a brief history (1–3 questions) AND (3) explains the session	No introduction	Only 1 out of the 3 introductory items	2 out of 3 introductory items	All 3 items of introductory items	0.489
Psychosocial Screening						
3	Asks the adolescent to talk without parent accompaniment and makes a confidentiality statement	None of these 2 items	-	1 of the 2 items	2 items	0.326
4	Conducts psychosocial screening related to weight management (home, eating, school, activity, stress)	No psychosocial screening	Screened only 1 issue of related psychosocial aspects	Screened 2 issues of related psychosocial aspects	Screened 3 or more issues of related psychosocial aspects	0.441
MI basic communication skills						
5	Open-ended questions	No open-ended questions	Some open-ended question(s), but no further probing	Open-ended questions followed by probing, but did not help the adolescent to talk about his/her difficulties/condition	Open-ended questions followed by probing and helped the adolescent talk more about his/her difficulties/conditions	0.662
6	Affirmation	No affirmation at all	Some affirmation, but was not suited to the context and did not focus on the patient’s strength or acknowledge their efforts	Some affirmation, that was well suited to the context, but did not focus on the patient’s strengths or acknowledge their efforts	Some affirmation, that was well suited to the context, and focused on the patient’s strengths or acknowledged their efforts	0.589
7	Reflective listening	No reflective listening	Some reflective listening, but was not suited to the context and did not lead to behavior change	Some reflective listening, that was well suited to the context, but did not lead to behavior change	Some reflective listening, that was well suited to the context and lead to behavior change	0.683
8	Making a summary	No summary provided at all	There was (some) summarizing but without patient’s confirmation and did not lead to behavior change	There was (some) summarizing, confirmed with the patient but did not lead to behavior change	There was (some) summarizing, confirmed with the patient and that lead to behavior change	0.386
MI stages						
9	Engaging	No engaging effort	Engaging effort, but did not suit the context of the conversation and did not lead to behavior change	Engaging effort, well suited to the context of the conversation, but did not lead to behavior change	Engaging effort, well-suited to the context of the conversation and that lead to behavior change	0.758
10	Focusing	No focusing effort	Some focusing effort, but did not suit the context of the conversation and did not lead to behavior change	Some focusing effort, well suited to the context of the conversation, but did not lead to behavior change	Some focusing effort, well-suited to the context of the conversation and that lead to behavior change	0,717
11	Evoking	No evoking effort	Some evoking effort, but was not suited to the context of the conversation and did not lead to behavior change	Some evoking effort, well suited to the context of the conversation, but did not lead to behavior change	Some evoking effort, well suited to the context of the conversation and that lead to behavior change	0.619
12	Planning	The patient was not ready for behavior change but the plan was mentioned by the health worker	The patient showed readiness for behavioral change but there was no guide or invitation to make a plan	The patient was not ready for a behavior change but the health worker was able to maintain an empathic and non-judgmental conversation	The patient actively participated in the planning discussion	0.462
13	Conveying the required information using the elicit-provide-elicit	Information was provided directly	Information was provided directly and was confirmed with the patient	Information was provided by asking the patient’s needs beforehand, but was not confirmed with the patient	Elicit-Provide-elicit was done completely	0,489
Involving Parents						
14	Parent involvement	Parents were not involved	Parents were involved but no discussion of parent roles	Discussed one dimension of parent role (structure OR support dimensions)	Discussed two dimensions of parent role (structure AND support dimensions)	0,018

### Construct validity and internal reliability

The mean value of the ICC measurement of the eight psychologists who rated 15 audio-taped counselling sessions was 0.931 (CI 0.868–0.973), indicating strong agreement. Using the 125 counselling audiotapes, the construct validation test of this assessment tool obtained a Cronbach value of 0.839. The correlation coefficient values were > 0.3 for all but two assessment items (items 1 and 14). To preserve the content validity of the final assessment tool, both items were retained after some minor revision to wording.

**Fig. 2:**
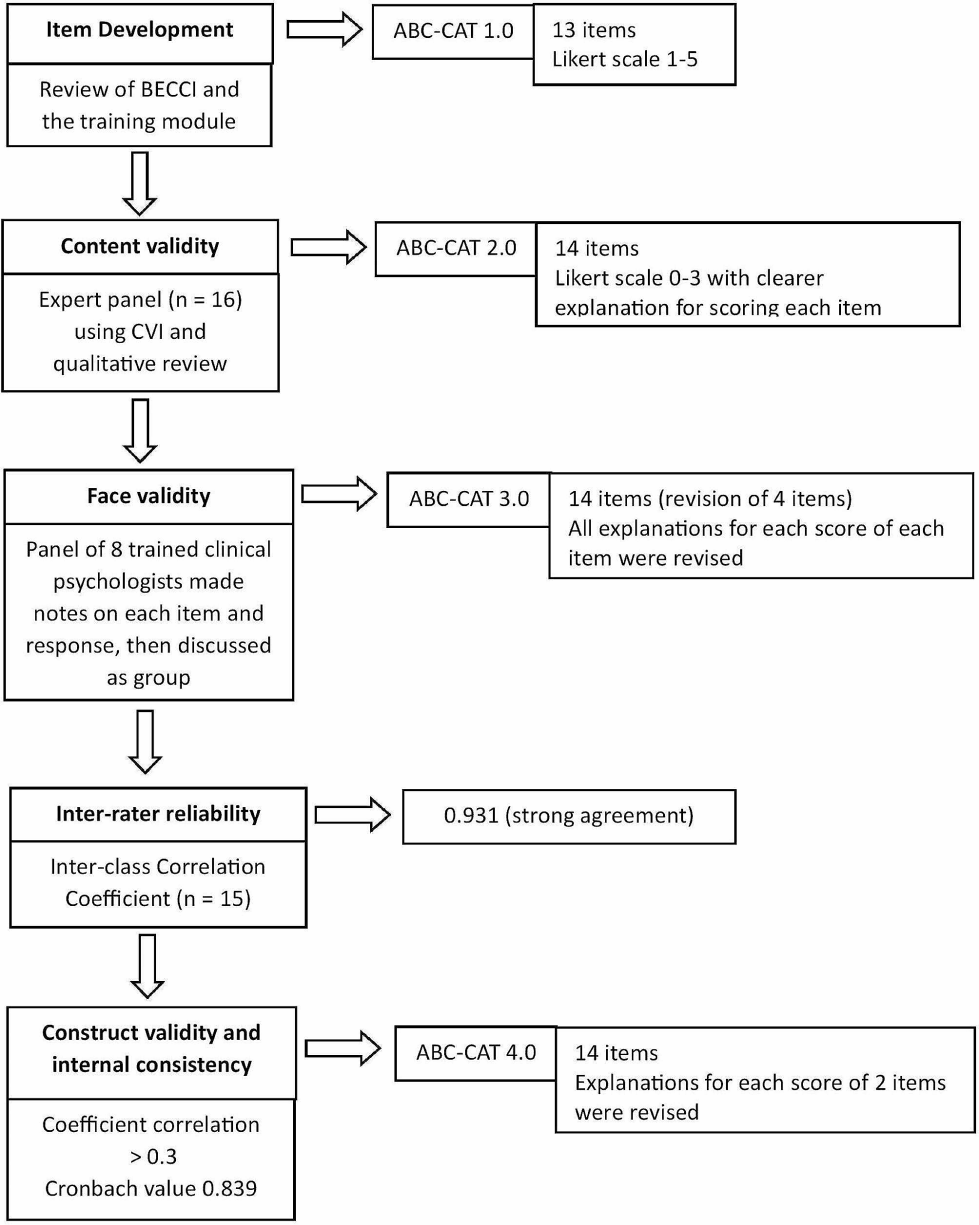
The processes and measurements tested with each version of the ABC-CAT

The overall process for developing the assessment tool is summarised in Fig. 2.

## Discussion

Within the tool, the assessment of four themes (the opening, psychosocial screening, specific behaviour-change counselling approach, and parental involvement) is consistent with our earlier identification of these themes as relevant for training in behavioural counselling. A focus on psychosocial screening is consistent with recommendations for counselling adolescents with behaviour-related health problems [16, 26, 27] and the foundational communication requirements with adolescent patients [28, 29]. Parental involvement is another distinctive element of this counselling training as parents critically influence adolescent growth and development, including adolescent behaviours [30, 31]. 

The careful development and validation process we undertook resulted in a series of changes to the assessment tool at each stage of its development. In particular, the face validity process resulted in changes to the scoring explanation of each item in response to evidence that our earlier explanations for scoring were at times unclear or ambiguous. This included changes to the Likert response scale, consistent with evidence that detailed, specific explanations of scoring for each item improves a tool’s performance [32, 33]. Each rater provided their reason for scoring each item, which informed the simplification of the scale from a 5-point to 3-point scale) and its response options, which ended up having more clear and concise definitions of each score for each item [33]. This is reflected in substantially better inter-rater reliability and internal consistency in the latter versions of the tool. Moreover, the performance level descriptions featured criteria that were more appropriate with the assessment’s purpose [34]. The final 14-item assessment tool revealed acceptable reliability (internal consistency) using Cronbach α scores. While cut-off values for questionnaire reliability are recognised to vary according to the field of application, a Cronbach α value ranging between 0.65 and 0.8 is typically considered adequate [35]. Comparison with other assessment tool is challenging due to differences with previously validated tools around the issues covered within the assessment tool, the number of items and type of questions, and the target population [36]. 

This assessment tool was used to assess behavioral change counselling for weight management in adolescents. While MI was initially developed for adults with substance use disorders, the application of MI has widely expanded to include other areas of behavior-related health and disease management such as adherence with medication for those with chronic diseases requiring long-term treatment adherence [16]. The general approach to MI has also expanded beyond clinical care to include preventive health measures such us developing healthy lifestyles and immunization programs [6, 16]. While MI was initially developed in adult populations, there is a large body of evidence about its relevance with adolescents [6, 37]. Other MI based assessment tools have not included specific items on the more generic aspects of counselling with adolescents. In particular, behaviour change for adolescents typically requires parent involvement due to their critical role in creating enabling home environments for healthy growth and development [13, 26, 27]. Notwithstanding its use in evaluating this training program that had a particular focus on weight, there is every expectation that this assessment tool could also be utilized to appraise the quality of counselling for other behavior-related health problems with adolescent patients.

A strength of this validation is that it was based on the assessments of clinical psychologists who understood basic communication skills with adolescent patients, the MI approach and the importance of involving parents in behavioral-change counselling with adolescents. The robust validity we have demonstrated no doubt reflects these skill sets. It is unknown to what extent less experienced professionals would achieve the same results, although it is anticipated that the development of an accompanying assessment guidance that includes real-life examples to guide evaluation may enhance consistent performance of the tool. Regardless, a current limitation is that the tool can only be used by trained raters who understand MI and the adolescent counselling principles covered in the assessment items.

## Conclusion

In sum, this study provides the first brief, feasible and validated assessment tool to evaluate health professionals’ skills in behaviour-change counselling for adolescent patients in Indonesia. Prior to expanding its use, the development of written guidance would be an important strategy to ensure the validity of future assessment. Future research is needed to assess the value of this assessment tool in research that aims to assess health professionals’ skills in working with adolescents on behavioral-change counselling beyond those relevant to overweight.

## Data Availability

Data and materials used for this study are available from the corresponding author upon request.

## Abbreviations

NCD: Non-communicable diseases
CVI: Content validity index
ICC: Inter-class correlation coefficient
MI: Motivational Interviewing
